# Two Distinct Clinical Phenotypes of Bulbar Motor Impairment in Amyotrophic Lateral Sclerosis

**DOI:** 10.3389/fneur.2021.664713

**Published:** 2021-06-16

**Authors:** Kaila L. Stipancic, Yana Yunusova, Thomas F. Campbell, Jun Wang, James D. Berry, Jordan R. Green

**Affiliations:** ^1^Speech and Feeding Disorders Lab, Department of Communication Sciences and Disorders, MGH Institute of Health Professions, Boston, MA, United States; ^2^UB Motor Speech Disorders Lab, Department of Communicative Disorders and Sciences, University at Buffalo, Buffalo, NY, United States; ^3^Speech Production Lab, Department of Speech-Language Pathology, University of Toronto, Toronto, ON, Canada; ^4^Speech, Language, Cognition, and Communication Lab, Department of Communication Sciences and Disorders, University of Texas at Dallas, Dallas, TX, United States; ^5^Speech Disorders and Technology Lab, Department of Communication Sciences and Disorders, University of Texas at Austin, Austin, TX, United States; ^6^Sean M. Healey and AMG Center for ALS, Massachusetts General Hospital, Boston, MA, United States

**Keywords:** amyotrophic lateral sclerosis, speech, dysarthria, phenotype, bulbar

## Abstract

**Objective:** Understanding clinical variants of motor neuron diseases such as amyotrophic lateral sclerosis (ALS) is critical for discovering disease mechanisms and across-patient differences in therapeutic response. The current work describes two clinical subgroups of patients with ALS that, despite similar levels of bulbar motor involvement, have disparate clinical and functional speech presentations.

**Methods:** Participants included 47 healthy control speakers and 126 speakers with ALS. Participants with ALS were stratified into three clinical subgroups (i.e., bulbar asymptomatic, bulbar symptomatic high speech function, and bulbar symptomatic low speech function) based on clinical metrics of bulbar motor impairment. Acoustic and lip kinematic analytics were derived from each participant's recordings of reading samples and a rapid syllable repetition task. Group differences were reported on clinical scales of ALS and bulbar motor severity and on multiple speech measures.

**Results:** The high and low speech-function subgroups were found to be similar on many of the dependent measures explored. However, these two groups were differentiated on the basis of an acoustic measure used as a proxy for tongue movement.

**Conclusion:** This study supports the hypothesis that high and low speech-function subgroups do not differ solely in overall severity, but rather, constitute two distinct bulbar motor phenotypes. The findings suggest that the low speech-function group exhibited more global involvement of the bulbar muscles than the high speech-function group that had relatively intact lingual function. This work has implications for clinical measures used to grade bulbar motor involvement, suggesting that a single bulbar measure is inadequate for capturing differences among phenotypes.

## Introduction

Amyotrophic lateral sclerosis (ALS) is a devastating neurodegenerative disease resulting in the progressive loss of limb, trunk, and head and neck (bulbar) motor function. The disease is characterized by significant across-patient heterogeneity in onset region, and pattern and rate of progression (1, 2). Understanding this heterogeneity in bulbar presentation is critical for improved understanding of disease symptomatology, as well as causes and implications of variability in expression, pathophysiology, and therapeutic response (3–6). Despite its complex and varied clinical manifestation (7), bulbar motor involvement in ALS has largely been considered a single variant, primarily graded on the severity of speech and swallowing symptoms (8).

The current work describes two clinical subgroups of patients with ALS who present with divergent profiles of bulbar motor involvement. Our goal was to determine if the two subgroups represented a single group that varied primarily in disease severity (i.e., single group hypothesis) or two distinct phenotypes of bulbar disease manifestation (i.e., two bulbar motor phenotype hypothesis). Overall, the findings support the existence of distinct phenotypes that, despite presenting with similar levels of bulbar motor involvement, exhibited disparate clinical and functional speech presentations.

## Methods

### Participants

Participants included 47 healthy control speakers (23 males, 24 females) and 126 speakers (73 males, 79 females), diagnosed with ALS by a neurologist following El Escorial criteria (8), from a larger study of bulbar impairment in ALS (9). All participants spoke English as their primary language; had no history of speech, language, hearing, or neurological problems (other than ALS); and had adequate vision and literacy skills to read stimuli. Symptom duration, expressed as patient report of months since symptom onset, and Amyotrophic Lateral Sclerosis Rating Scale-Revised (ALSFRS-R); (10) total and bulbar subscores were used to compare overall disease severity, as well as bulbar specific severity, between the participant groups.

### Standard Protocol Approvals, Registrations, and Patient Consents

This study was approved by the Institutional Review Boards at the Mass General Brigham (MGB), University of Nebraska, University of Toronto, and University of Texas at Dallas. Written informed consent was obtained from all participants prior to being enrolled in the study.

### Procedure

Data were collected across multiple sites. Participants completed a standard research protocol designed to capture both clinical impairment (i.e., speech intelligibility, oral mechanism function, etc.) and instrumentation-based measures of speech motor impairment (11). The Speech Intelligibility Test (SIT) (12) was used to stratify participants into high and low speech-function subgroups (described below). Multiple speech outcome variables were derived from (1) a standardized paragraph reading passage to characterize patterns of continuous speech, and (2) a rapid syllable repetition task, a more challenging task for testing the speed generating capacity of oral muscles. To maximize data yield, we included all the available data for each experimental task for each participant, even though some participants did not complete all tasks. Additionally, we included data for as many healthy control speakers as was available. The number of participants included for each analysis is displayed in Table 1, along with outcome measures derived from each task, which are further described below.

**Table 1:** Outcome measures derived from each task and number of participants included in each analysis.

**Task**	**Data analysis**	**Measures**	**No. of participants included in the analysis**
Sentence reading task [Speech Intelligibility Test (SIT) (12)]	Orthographic transcription by research assistant	Speech intelligibility (%)	173
	Number of words/total duration	Speaking rate (SR; WPM)	173
Paragraph reading task (Bamboo passage)	Speech pause analysis (SPA) (13)	Percent pause (%)	150
		Articulation rate (AR; syllables/s)	150
	Acoustic analysis—formant tracking of F2 in “flower” conducted in Praat (14)	F2 range (Hz)	119
Rapid syllable repetition task	Automatic extraction of lip movement (15)	Maximum velocity (mm/s)	63
		Duration (s)	63

*F2, second formant; Hz, hertz; mm, millimeters; s, seconds; WPM, words per minute*.

### Tasks and Outcome Measures

#### Sentence Reading Task

A sentence reading task (SIT) (12) was administered following a standard research protocol (11, 16–19). Participants were audio-recorded while reading aloud, at their typical rate and loudness, 11 randomly generated sentences ranging from 5 to 15 words in length. Trained research assistants orthographically transcribed the recorded sentence productions offline. Percent intelligibility for each sentence was calculated (number of correctly transcribed words/number of target words × 100) and averaged across the 11 sentences to derive an overall speech intelligibility score for each participant. Speaking rate (SR) was calculated for each sentence as the number of words produced divided by the total duration (including pause intervals). SR was averaged across the 11 sentences to derive each participant's overall SR in words per minute (WPM). Strong intra- and inter-rater reliability has previously been reported for SR with correlations of 0.93 and intraclass correlation coefficients (ICCs) of 0.53 (18, 20).

#### Paragraph Reading Task

**Figure 1:**
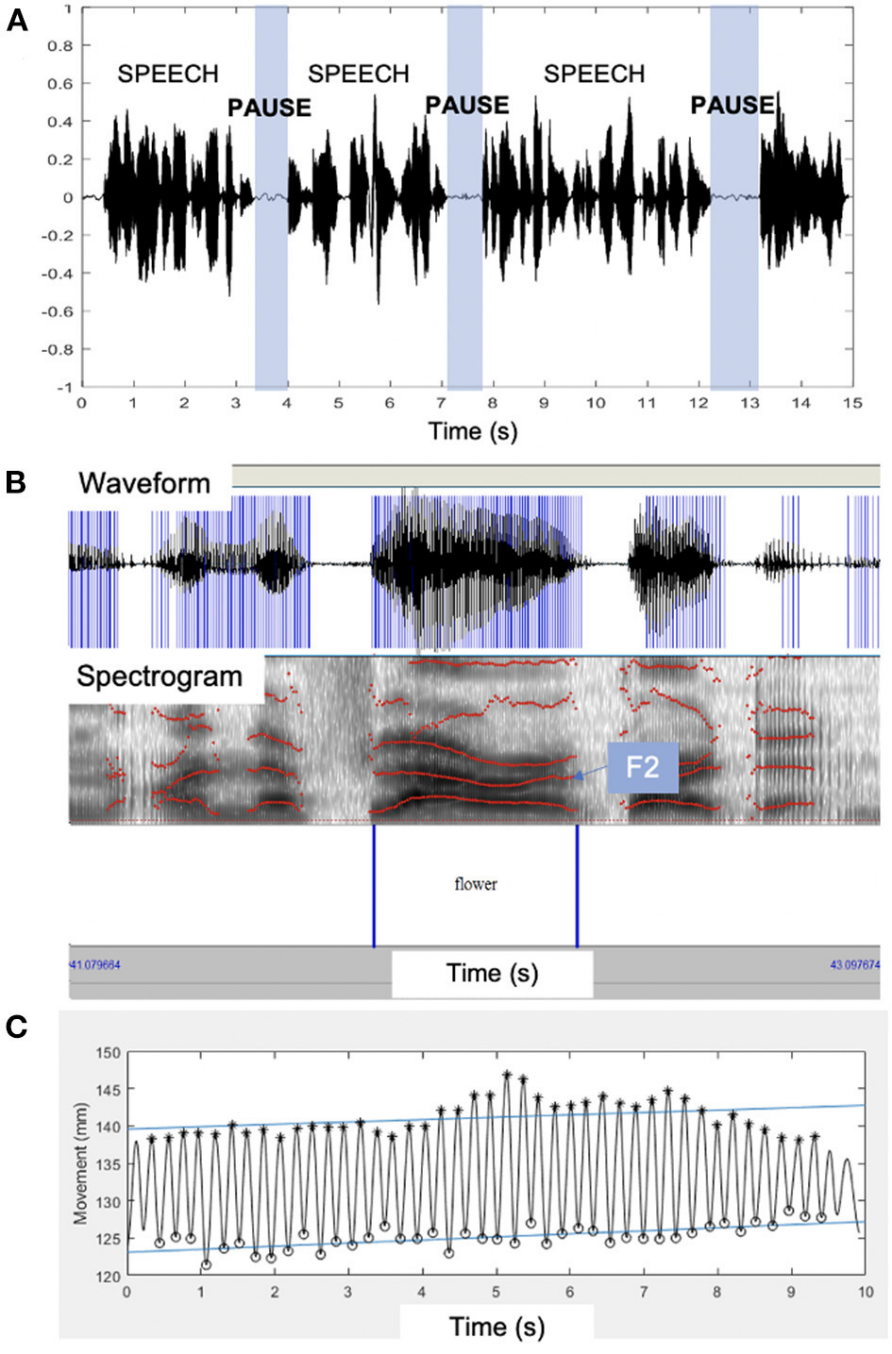
Examples of the quantitative speech measures used in this paper. **(A)** A rendering of speech pause analysis (SPA)^18^ of the acoustic waveform with speech and pause segments marked over time. **(B)** The acoustic waveform and spectrogram with the second formant (F2) marked on the spectrogram from which F2 range was extracted. **(C)** Kinematic signal derived from lip movements during the rapid syllable repetition task. F2, second formant; s, seconds.

Participants were instructed to read aloud a short 98-word paragraph, the Bamboo Passage (21), at their typical rate and loudness. Paragraph productions were recorded and later analyzed with speech pause analysis (SPA) (13), a custom MATLAB routine designed to extract SR and pausing metrics. Specifically, we extracted percent pause and articulation rate (AR; excluding pause intervals) in syllables/second (20). A rendering of the SPA analysis is displayed in Figure 1A. Moderate intra- and inter-rater reliability have previously been reported for these measures with ICCs ranging from 0.49 to 0.61 (20).

In addition, the second formant (F2) range during the segment “flower” was extracted using the acoustic analysis software Praat (14). Formants are vocal tract resonances that are primarily driven by the movement of oral structures. F2 is associated with anterior-posterior tongue movement in the mouth (22, 23) and its range (maximum–minimum) is considered a proxy for tongue movement (24–26), with greater values indicating more typical movement. We created a custom Praat script to extract the F2 values across the hand-marked segment “flower.” This segment was chosen to maximize the range of F2, as production of the diphthong (“ow”) necessitates considerable tongue movement. See Figure 1B for an example analysis of F2 range.

#### Rapid Syllable Repetition Task

Movement of the lips (kinematics) were recorded during a rapid syllable repetition task, known as the alternating motion rate (AMR) task. Participants were asked to take a deep breath and repeat the syllable “bah” as quickly and accurately as possible, for as long as possible, on one breath. This AMR task is a maximum performance task (27, 28) that tests the speed-generating capacity of the lips and jaw. Electromagnetic articulography (Wave; Northern Digital, Inc.) was used to track three-dimensional lip movement during the task. A six-degree-of-freedom (6DOF) sensor was placed on the head to subtract head movement from the lip movement. 5DOF sensors were individually placed on the upper and lower lips using medical tape. Movement traces were analyzed in MATLAB and semi-automatic, algorithmic extraction of lip movement was performed (15). The algorithm provided 21 features of lip movement during the rapid syllable repetition task. We used two articulatory parameters shown to be sensitive markers of disease progression (15, 28, 29): maximum velocity (mm/second) of lower lip movement (maximum velocity across the entire production) and total duration (seconds) of the syllable repetition task. An example of the lip movement analysis is displayed in Figure 1C. A single analyst extracted these measures because they are algorithmically derived and fully replicable (15).

### Participant Stratification

Previous work has identified SR as a sensitive marker of speech impairment (11, 17, 20). Thus, we divided participants into three groups using a cutoff SR of 150 WPM derived from the SIT, for speakers with and without bulbar motor impairment, in accordance with prior studies (30, 31). Speech intelligibility was considered a measure of speech function as it putatively reflects overall communication effectiveness (32). A 96% intelligibility cutoff was chosen based on previous work finding a minimally detectable change of ~3% intelligibility for speakers with near-normal levels of intelligibility (18). The three primary group divisions were as follows: Table 2 displays the stratification criteria for each group:

1. *Healthy control speakers* had no evidence of speech motor impairment (based on intelligibility and SR criteria);
2. *Bulbar asymptomatic speakers* were diagnosed with ALS, but had minimal to no evidence of bulbar impairment; and
3. *Bulbar symptomatic speakers* were diagnosed with ALS and had evidence of bulbar impairment.

**Table 2:** Demographic information of participants.

	**Healthy controls**	**Bulbar asymptomatic**	**Bulbar symptomatic**		***p-*values**
			**High speech function**	**Low speech function**	
**Stratification criteria**	No history of speech, language, or neurological problems	Diagnosed with ALS; speaking rate >150 WPM; intelligibility >96%	Diagnosed with ALS; speaking rate <150 WPM; intelligibility >96%	Diagnosed with ALS; speaking rate <150 WPM; intelligibility <96%	N/A
**Total** ***N*** **[number of males (M)]**	47 (23 M)	60 (33 M)	38 (20 M)	28 (20 M)	N/A
**Mean age in years (SD)**	60.80 (8.47)	59.53 (10.22)	56.74 (8.51)	59.63 (9.92)	All groups: *p* = 0.25
**Symptom duration—mean months since symptom onset (SD)**	N/A	42.63 (27.95)	42.78 (43.02)	36.00 (33.11)	All ALS groups: *p* = 0.70
**Site of Onset =** ***N***	N/A	Spinal = 48 Bulbar = 1 Mixed = 3 Unknown = 8	Spinal = 30 Bulbar = 4 Mixed = 0 Unknown = 4	Spinal = 17 Bulbar = 7 Mixed = 2 Unknown = 0	N/A
**Mean ALSFRS-R Total (SD)**	N/A	32.45 (8.07)	32.10 (8.25)	33.38 (10.27)	All ALS groups: *p* = 0.87
**Mean ALSFRS- R Bulbar (SD)**	N/A	11.06 (1.42)	9.77 (1.76)	8.55 (2.82)	All ALS groups: *p* < 0.001
					High speech-function and low speech-function groups: *p* = 0.07
**Mean % Intelligibility (SD)**	99.25 (1.16)	99.15 (1.03)	98.44 (1.46)	71.61 (29.93)	All ALS groups: *p* < 0.001
					High speech-function and low speech-function groups: *p* < 0.001
**Mean speaking rate in WPM (SD)**	186.88 (21.61)	182.65 (18.58)	115.85 (24.24)	109.20 (24.68)	All ALS groups: *p* < 0.001
					High speech-function and low speech-function groups: *p* = 0.61

*ALS, Amyotrophic lateral sclerosis; ALSFRS-R, Amyotrophic lateral sclerosis rating scale-revised; SD, standard deviation; WPM, words per minute*.

*p-values: derived from ANOVAs between participant groups*.

***Note:** Stratification criteria were defined using currently accepted metrics of bulbar impairment based on previous literature, which is described in detail in the text*.

***Note:** In the bulbar asymptomatic group, mean ALSFRS-R bulbar subscore was <12, indicating that at least a few participants reported some level of bulbar function impairment. However, we defined bulbar impairment on the clinical measures (i.e., speaking rate and speech intelligibility) used; we kept all participants who met these criteria in the bulbar asymptomatic group. All participants in this group were, therefore, very early on in disease progression and were asymptomatic based on our clinical metrics of speech*.

Within the bulbar symptomatic group, we identified a potential stratification into two subgroups based on highly disparate clinical presentations of functional speech (i.e., speech intelligibility):

- Bulbar symptomatic speakers with *high speech function* had evidence of bulbar impairment but preserved speech function; and
- Bulbar symptomatic speakers with *low speech function* had evidence of bulbar impairment and degraded speech function.

### Statistical Analyses

We examined differences between the groups in each of the outcome variables, as well as demographic variables, using analysis of variance (ANOVA) models. *Post hoc* tests (Tukey's HSD) were conducted for statistically significant main effects. All statistical analyses were completed in R (33).

## Results

### Group Stratification Variables

Differences in SR and intelligibility between the subgroups were by design. However, the statistical differences between groups are presented here to provide validation of our stratification scheme. These data are also displayed in Table 2. SR was significantly different between all groups [*F*_(3, 169)_ = 147.6, *p* < 0.001], except between healthy controls (mean = 186.88, SD = 21.61) and bulbar asymptomatic speakers (mean = 182.65, SD = 18.58, *p* = 0.75), and between the high (mean = 115.85, SD = 24.24) and low speech-function groups (mean = 109.20, SD = 24.68, and *p* = 0.61). There was a main effect of group for speech intelligibility [*F*_(3, 169)_ = 40.7, *p* < 0.001], and despite having comparable SR, the high (mean = 98.44, SD = 1.46) and low speech-function groups (mean = 71.61, SD = 29.93) differed significantly in speech intelligibility (*p* < 0.001). The healthy controls (mean = 99.25, SD = 1.16), bulbar asymptomatic group (mean = 99.15, SD = 1.03), and high-speech function group did not differ in speech intelligibility (*p* > 0.05).

### Group Differences in Clinical Bulbar Metrics

Table 2 shows the demographic information of the participants in the four proposed groups. Symptom duration [*F*_(2, 113)_ = 0.362, *p* = 0.70] and ALSFRS-R total score [*F*_(2, 99)_ = 0.142, *p* = 0.868] were not significantly different between any of the groups with ALS (*p* = 0.71–1.00, *p* = 0.86–0.98, respectively). Mean ALSFRS-R bulbar subscore [*F*_(2, 99)_ = 14.18, *p* < 0.001] was significantly higher (i.e., less impaired) for the bulbar asymptomatic group than the two bulbar impaired groups (*p* < 0.05); however, the bulbar subscore did not differ between the two impaired groups (*p* = 0.066).

### Group Differences in Passage Reading Task

Significant main effects of group were found for percent pause [*F*_(3, 146)_ = 9.32, *p* < 0.001] and AR [*F*_(3, 126)_ = 32.58, *p* < 0.001]. The high (percent pause mean = 23.85, SD = 7.28; AR mean = 3.64, SD = 0.87) and low speech-function groups (percent pause mean = 24.37, SD = 9.38; AR mean = 3.66, SD = 0.69) did not differ significantly from each other in either percent pause (*p* = 0.99) or AR (*p* = 1.00), although both groups significantly differed from healthy controls (pause mean = 16.39, SD = 6.62, *p* < 0.001; AR mean = 5.09, SD = 0.50, *p* < 0.001, respectively). The bulbar asymptomatic group did not differ from any group on speaking rate (mean = 19.81, SD = 6.33, *p* > 0.05) but had a significantly faster articulation rate (mean = 4.67, SD = 0.87) than both the high speech-function group (*p* < 0.001) and the low speech-function group (*p* < 0.001).

**Figure 2:**
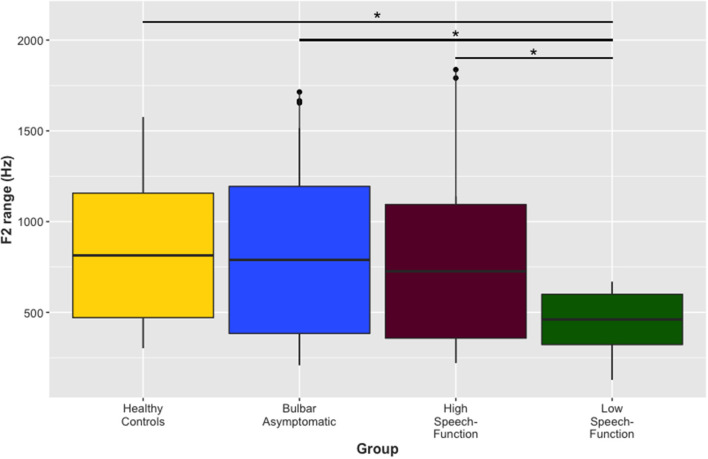
Range of the second formant (F2). Range of F2 (Hz) in the segment “flower” from the passage reading task across the four groups. Healthy controls, the bulbar asymptomatic group, and the high speech-function group did not differ from each other, but the low speech-function group had a significantly smaller F2 range than the three other groups. [*p < 0.05]. F2, second formant; Hz, hertz.

There was also a main effect of group for F2 range (in Hz) in the segment “flower” from the passage reading task [*F*_(3, 115)_ = 4.87, *p* = 0.003]. Figure 2 shows the data for each group on this variable. Healthy controls (mean = 814.04, SD = 342.87), bulbar asymptomatic (mean = 789.21, SD = 405.09), and the high speech-function group (mean = 726.30, SD = 368.30) did not differ from each other (*p* > 0.05), but all significantly differed from the low speech-function group (mean = 461.29, SD = 138.58, *p* < 0.05) which had the smallest F2 range.

### Group Differences in Rapid Syllable Repetition Task

Results also revealed significant main effects of group in the two kinematic features extracted from the syllable repetition task {maximum velocity [*F*_(3, 59)_ = 4.407, *p* = 0.007] and duration [*F*_(3, 59)_ = 7.529, *p* < 0.001]}. Bulbar asymptomatic speakers (mean = 232.58, SD = 72.57) had higher maximum velocities in lip movement than both the high (mean = 162.54, SD = 70.61, and *p* = 0.02) and low speech-function groups (mean = 154.01, SD = 54.74, and *p* = 0.01), but the latter two groups did not differ from each other (*p* = 0.98). Healthy controls had a longer duration (mean = 21.21, SD = 7.50) of syllable repetition task than each of the other three groups (bulbar asymptomatic, mean = 14.90, SD = 6.15, and *p* = 0.04; high speech-function group, mean = 11.77, SD = 6.49, and *p* < 0.001; low speech-function group mean = 12.59, SD = 4.85, and *p* = 0.002); however, none of the latter three groups differed significantly from each other (*p* > 0.05).

## Discussion

The purpose of this study was to examine potential phenotypes of bulbar motor involvement in individuals with ALS based on clinical stratifications of individuals with similar levels of bulbar involvement, but disparate speech function profiles. To determine if the two bulbar symptomatic groups represented two distinct phenotypes of bulbar impairment vs. a single phenotype that varied in severity, we tested for group differences in indices of overall bulbar motor severity and symptom duration. If differences in speech function were primarily due to differences in disease stage or severity (i.e., and support the single group hypothesis), then we expected the high speech-function group to have higher (i.e., less impaired) scores on indices of overall ALS severity (i.e., ALSFRS-R total scores) and bulbar motor severity [i.e., ALSFRS-R bulbar subscore, speaking rate (SR), articulation rate (AR), percent pause, and maximum lip movement velocity], as well as a shorter symptom duration, than the low speech-function group.

Our findings did not provide evidence for the single group hypothesis. First, the acoustically derived proxy for tongue movement, range of F2 (34), differed between the two groups with bulbar impairment, with the high speech-function group (who had intact speech intelligibility) appearing to exhibit much greater lingual movement than the low speech-function group (who had degraded speech intelligibility). Second, both subgroups were statistically equivalent on most indices of overall ALS and bulbar motor severity. Consistent with prior findings on neurodegenerative disorders of speech (16, 20), both bulbar impaired subgroups used a greater percentage of pause and a slower rate of articulation, and had shorter durations on the rapid syllable repetition task, than did healthy controls and bulbar asymptomatic speakers. The absence of detectible differences among the two bulbar symptomatic groups on these clinical severity measures is unlikely due to a Type II error from measurement imprecision, as the measures' responsiveness to bulbar motor decline has been previously demonstrated (17, 20, 35). Moreover, the large effects in measures between the control groups (i.e., healthy controls and bulbar asymptomatic) and bulbar symptomatic subgroups (i.e., high and low speech-function groups) provided additional support for the assertion that the absence of differences across bulbar symptomatic groups was not due to measurement error (i.e., inaccuracy or imprecision).

The two-phenotype hypothesis was further supported by findings suggesting that slowed speech in the high speech-function group was due to primary disease effects rather than a behavioral adaption intended to preserve speech function. Rate reduction is a common compensatory response to not being understood and often has the effect of optimizing speech clarity and intelligibility (36). Slow speech affords additional time to clearly articulate speech sounds (26, 37, 38) and to coordinate the speech subsystems (38, 39), including optimizing breath placement. It was, therefore, possible that the slowed speech observed in the high speech-function group was an adaptive response to improve speech clarity (40, 41) rather than an ALS-related constraint on bulbar neuromuscular function. Our findings, however, did not support the use of adaptive strategy because (1) the high speech-function group did not show evidence of rate-slowing strategies such as such increased pausing (16) or reduced articulation rate (12) that differed from the low speech-function group, and (2) our testing of jaw and lip muscle speed generating capacity on the syllable repetition task revealed a similar neuromuscular constraint on speed of articulatory movements in both groups (42).

Overall, our findings provided evidence that phenotypic variation may be marked by the difference in the regionality of involvement within the bulbar musculature. More specifically, the speech patterns of the low speech-function group were consistent with more global involvement of the bulbar muscles than that of the high speech-function group, which, by comparison, appeared to have relatively intact lingual motor function. Findings from syllable repetition, pausing, and duration analyses did not support differences in respiratory function between the bulbar impaired groups. Although measures of pausing patterns in speech have been used to index respiratory muscle involvement (21), pauses are also affected by other factors such as cognition (20); therefore, future work could benefit from the inclusion of more direct measures of respiratory function, such as functional vital capacity. Additionally, the rate of lip and jaw movements, as tested by the rapid syllable repetition task, was not different between the bulbar impaired groups. Given the importance of tongue movement to speech intelligibility (5), it was not unexpected that tongue involvement would distinguish the low and high speech-function groups (43). Because tongue function during speech was measured indirectly based on the acoustically derived F2 range, future work could benefit from more direct measures such as biomechanic analyses of tongue movement using electromagnetic articulography or ultrasound (44).

Prior research on the spread of motor signs and symptoms in the spinal system has shown wide variation in the muscles that are first affected, as well as the pattern of spread from upper to lower motor neurons and from muscle to muscle (1, 45–47). Similar heterogeneity would be expected among bulbar muscles because of the somatotopic organization of primary motor pathways and because muscles are innervated by distinct cranial nerves (i.e., lip, jaw, and tongue innervated by cranial nerves VII, X, and XII, respectively) with nuclei distributed throughout the brainstem. These findings challenge early work suggesting that the tongue is the leading indicator of bulbar impairment (7, 48), as the high speech-function group in the current study did not conform to this pattern. We hypothesize that impaired tongue control can manifest differently in different patients with ALS, which may depend on focality and spread of motor neuron damage.

### Clinical/Research Implications and Future Directions

The current work has implications for clinical outcome measures used to grade bulbar motor involvement, demonstrating that using a single indicator of bulbar motor involvement may belie the phenotypic complexity of patients with bulbar involvement, significantly affecting the quality of clinical research and treatment of patients with ALS. Acknowledging putative bulbar motor phenotypes may have implications for speech outcomes that are used in both clinical and research settings. Future work is needed to understand how these groups of speakers with disparate clinical presentations differentially respond to a variety of therapeutic speech approaches, including using a bite block to provide jaw stabilization during speech, speaking modifications such as clear or loud speech (49), and pharmacological treatments. Future longitudinal studies could provide information about the progression of symptomatology to further elucidate the neural, biomechanical, and behavioral mechanisms that account for the phenotypic variations inferred from the current data.

### Study Limitations

Given that the dataset included a sample of data collected across three labs over ~10 years, we were unable to include measures that provide information about each individual articulator. We were also unable to account for the status of each speech subsystem or other patient-related factors, such as cognitive function, medications, respiratory status (i.e., vital capacity), etc. Lastly, there was a selection bias in the design of this study, which limits our ability to make conclusions about prevalence of these phenotypes among all patients with ALS, and there may exist additional phenotypes of bulbar motor impairment unexamined in the current study. Future studies involving a representative sample of individuals with ALS are needed to further validate the proposed phenotypes and provide detailed information about proportions of the population within each phenotype.

## Data Availability Statement

The anonymized data will be made available by the authors upon reasonable request from any qualified investigator.

## Ethics Statement

The studies involving human participants were reviewed and approved by the Mass General Brigham (MGB), University of Nebraska, University of Toronto, and University of Texas at Dallas. The patients/participants provided their written informed consent to participate in this study.

## Author Contributions

KS had a major role in study conceptualization and design, data analyses, interpretation of the findings, and writing of the manuscript. YY, TC, JW, and JB contributed to the interpretation of the findings, reviewed the manuscript, and provided feedback. JG had a major role in the study conceptualization and design, interpretation of the findings, and manuscript preparation. All authors contributed to the article and approved the submitted version.

## Conflict of Interest

The authors declare that the research was conducted in the absence of any commercial or financial relationships that could be construed as a potential conflict of interest.
